# Overcoming Obstacles to Targeting Muscarinic Receptor Signaling in Colorectal Cancer

**DOI:** 10.3390/ijms22020716

**Published:** 2021-01-13

**Authors:** Osman Ali, Mazen Tolaymat, Shien Hu, Guofeng Xie, Jean-Pierre Raufman

**Affiliations:** 1 Department of Medicine, Division of Gastroenterology & Hepatology, University of Maryland School of Medicine, Baltimore, MA 21201, USA; oali@som.umaryland.edu (O.A.); mtolaymat@som.umaryland.edu (M.T.); shu1@som.umaryland.edu (S.H.); gxie@som.umaryland.edu (G.X.); 2 Veterans Affairs Maryland Healthcare System, Baltimore, MA 21201, USA; 3 Marlene and Stewart Greenebaum Comprehensive Cancer Center, University of Maryland School of Medicine, Baltimore, MA 21201, USA; 4 Department of Biochemistry and Molecular Biology, University of Maryland School of Medicine, Baltimore, MA 21201, USA

**Keywords:** muscarinic receptors, colorectal cancer, matrix metalloproteinases, acetylcholine, epidermal growth factor receptors

## Abstract

Despite great advances in our understanding of the pathobiology of colorectal cancer and the genetic and environmental factors that mitigate its onset and progression, a paucity of effective treatments persists. The five-year survival for advanced, stage IV disease remains substantially less than 20%. This review examines a relatively untapped reservoir of potential therapies to target muscarinic receptor expression, activation, and signaling in colorectal cancer. Most colorectal cancers overexpress M_3_ muscarinic receptors (M_3_R), and both in vitro and in vivo studies have shown that activating these receptors stimulates cellular programs that result in colon cancer growth, survival, and spread. In vivo studies using mouse models of intestinal neoplasia have shown that using either genetic or pharmacological approaches to block M_3_R expression and activation, respectively, attenuates the development and progression of colon cancer. Moreover, both in vitro and in vivo studies have shown that blocking the activity of matrix metalloproteinases (MMPs) that are induced selectively by M_3_R activation, i.e., MMP1 and MMP7, also impedes colon cancer growth and progression. Nonetheless, the widespread expression of muscarinic receptors and MMPs and their importance for many cellular functions raises important concerns about off-target effects and the safety of employing similar strategies in humans. As we highlight in this review, highly selective approaches can overcome these obstacles and permit clinicians to exploit the reliance of colon cancer cells on muscarinic receptors and their downstream signal transduction pathways for therapeutic purposes.

## 1. Introduction

Colorectal cancer (CRC) is the second and third most commonly occurring cancer in women and men, respectively; approximately 1.8 million new cases were reported worldwide in 2018 [1]. Most cases occur in industrialized countries with high consumption of so-called ‘western diets’ [2]. In the United States (U.S.), it is estimated that 147,950 persons were diagnosed with CRC in 2020, comprising 104,610 colon and 43,340 rectal cancers. Although CRC is predominantly diagnosed in persons 50 years and older, the increasing prevalence of CRC in younger persons, particularly African American men, is concerning; it is estimated that 17,930 new cases of CRC will occur in persons younger than 50 years [3]. Overall, in the U.S. in 2020 it is anticipated 52,300 deaths will be attributed to CRC [3,4]. Despite advances in screening and public health initiatives to increase CRC awareness and the importance of screening, CRC remains a leading cause of cancer morbidity and mortality. It is particularly concerning that despite decreasing rates in those older than 55 years, for currently unknown reasons, the incidence of metastatic CRC is increasing at an alarming rate in those who are younger [5,6].

The management of CRC depends on tumor stage, location, and other patient-specific characteristics. Surgical excision remains the mainstay of therapy for early-stage CRC; however, treatment options for advanced metastatic disease remain focused on adjuvant or neoadjuvant chemotherapy, depending on whether the individual meets criteria for surgery [7]. Based on evidence of improved response rates and progression-free survival, first-line chemotherapy for metastatic CRC (mCRC), e.g., capecitabine and 5-fluorouracil (5-FU), is commonly combined with other cytotoxic agents such as irinotecan or oxaliplatin [8]. Nevertheless, despite improved treatment strategies the majority of patients with advanced mCRC succumb to disease within five years as a consequence of initial or acquired drug resistance and lack of efficacy [9].

The principal mechanism underlying resistance to chemotherapy and subsequent cancer progression is altered programmed cell death (apoptosis), which is controlled by intricate signaling pathways. Chemotherapeutic regimens such as FOLFOX (5-FU/leucovorin/oxaliplatin) and FOLFOXIRI (5-FU/leucovorin/oxaliplatin/irinotecan) generally promote apoptosis by killing susceptible cells and allowing resistant cells to proliferate and repopulate tumors [7]. Thus, targeting the mechanisms that promote cell survival (i.e., resistance to apoptosis) has become a promising strategy to treat mCRC; this is largely accomplished by combining FOLFOX or FOLFOXIRI with monoclonal antibodies and kinase inhibitors targeting specific survival pathways, e.g., those downstream of epidermal and vascular endothelial growth factor receptors (EGFR and VEGF) [10]. Nonetheless, even these advances generally provide limited response rates, measured in months gained, and mCRC continues to have a dismal five-year survival in the range of 14.3 % [11].

Novel immunotherapeutics have shown great benefit for some cancers, but their efficacy for mCRC appears limited to those with sporadic or inherited mismatch repair defects. Overall, it is estimated that fewer than 10% of patients with mCRC will derive benefit from immune checkpoint inhibitors and, even then, responses in a small subset of patients are limited to only months of added life [12,13].

To address these challenges, newer approaches to target key signaling pathways that mediate cancer cell proliferation, survival, invasion, and metastasis are imperative. For example, as mutations in receptor or downstream targets such as c-MYC, EGFR, KRAS, NRAS, BRAF, PTEN, or SMAD2/4 confer resistance to therapy and shorter survival, major efforts are underway to overcome these obstacles to successful therapy [14]. In addition to genetic mutations, alterations in non-coding RNAs, e.g., microRNAs, that mediate post-transcriptional regulation of protein expression may alter tumor progression and serve as both prognostic biomarkers and therapeutic targets. While further treatments are being developed and tested for efficacy, a better understanding of disease pathobiology is likely to identify novel therapeutic targets [15].

In this context, the M_3_ subtype cholinergic muscarinic receptor (M_3_R) was identified as a promotor and regulator of colon cancer cell progression. M_3_R activation promotes CRC progression by both EGFR-dependent and -independent mechanisms [16]. Elucidating the molecular mechanisms underlying these pathways has already provided valuable insights into potential downstream targets. Here, we review current knowledge regarding how muscarinic agonists activate M_3_R, downstream signaling, and kinase activation, and how these actions result in disease progression. Importantly, we consider how these advances in knowledge may identify novel targets and consider current obstacles to leveraging these targets therapeutically. 

## 2. Muscarinic Receptor Subtypes in Normal Physiology

The seven transmembrane domain G protein-coupled receptor (GPCR) muscarinic receptor family comprises five subtypes, designated M_1_R to M_5_R, that are expressed in a wide variety of organs and tissues [17,18]. Consequently, these receptors regulate a variety of important biological functions including ion channel transport, smooth muscle contraction, lipid turnover, and adenylyl cyclase activity [19,20,21,22]. There is an extensive sequence homology within the seven transmembrane spanning domains of these receptors [23]. Sequence variability is exhibited at the extracellular amino terminus and within the third intracellular loop [24]. The selectivity of G protein coupling depends on the individual muscarinic receptor, defined by amino acids within the second intracellular loop and further in the membrane portion of the third loop [24]. The physiological activity of muscarinic receptors depends on tissue expression, G protein coupling, and the downstream signal transduction pathway. In general, stimulation of odd-numbered muscarinic receptors, M_1_R, M_3_R, and M_5_R, is coupled to activation of G proteins in the G_q11_ family and downstream activation of phospholipase C-β and phosphatidylinositol trisphosphate turnover that enhances calcium flux. The even-numbered muscarinic receptors, M_2_R and M_4_R, are generally coupled to G proteins within the G_i/o_ family, and their stimulation inhibits adenylyl cyclase activity, thereby reducing levels of intracellular cAMP [25]. 

Although muscarinic receptor expression may be ubiquitous, patterns of subtype distribution vary from organ to organ and receptor subtypes may have overlapping, or distinct functions. For example, both the brain and the eye express all five muscarinic receptor subtypes; however, M_5_R predominates in the brain, whereas M_3_R predominates in the eye [26,27,28,29,30,31]. M_1_R is widely distributed in the heart, uterus, GI tract, and central nervous system. In the heart, M_1_R activation increases calcium concentration and stimulates tachycardia, whereas M_2_R activation results in bradycardia [32,33]. Activation of M_3_R relaxes systemic vascular tone [34] while M_5_R activation relaxes cerebral vascular tone [35]. Within the gut, pulmonary airways [36], bladder [37], uterus [38], and smooth muscle, M_1_R, M_2_R, and M_3_R are the predominantly expressed and studied muscarinic receptor subtypes [39]. Whereas M_1_R and M_3_R appear to play a key role in gut epithelial cell function, M_2_R appears to be more important for subepithelial smooth muscle cell function. For example, M_3_R regulates acid secretion from gastric parietal cells, and a mix of M_1_R and M_3_R regulate pepsinogen release from gastric chief cells [40,41,42]. Nevertheless, even these functions may be complicated by interactions between the muscarinic receptor subtypes. Therefore, while smooth muscle cell function per se may be regulated by M_2_R activity, overall smooth muscle tone is regulated tightly by the autonomic and enteric nervous systems that signal via M_1_R and M_3_R [43]. 

## 3. M_3_R Expression in CRC 

The M_3_R muscarinic receptor subtype, encoded by *CHRM3*, plays a prominent role in CRC progression. *CHRM3* is a conditional oncogene whose expression stimulates cell proliferation and invasion, resistance to apoptosis, and, in general, cell functions that result in the progression of CRC and metastasis [44,45]. Most colon cancers overexpress M_3_R/*CHRM3* [44,46,47,48]. Likewise, several human colon cancer cell lines commonly used in biomedical research, e.g., HT-29 and H508 cells, overexpress M_3_R/*CHRM3* [44,49]. Compared to normal colon tissues, Yang et al. detected eight-fold greater *CHRM3* RNA expression in colon cancer specimens [45]. Experiments inhibiting M_3_R activity in HT-29 cells [46] or comparing M_3_R expression in CRC to normal colon tissue [44] confirmed the impact of M_3_R/*CHRM3* expression and activity on CRC progression. 

In the normal colon, relatively weak M_3_R expression is restricted primarily to basolateral membranes of surface epithelial cells. However, in CRC, M_3_R is expressed diffusely along cell membranes, consistent with the loss of cell polarity in neoplasia [44]. Interestingly, although there is a significant association between the level of M_3_R/*CHRM3* expression in primary tumors and the presence of CRC metastases, M_3_R/*CHRM3* expression within metastases is not increased, suggesting M_3_R/*CHRM3* overexpression is less important and impactful for cancer cell function once CRC cells have metastasized [44]. It would be of great interest to uncover and possibly leverage the biological cues and signaling programs that lead to and mediate this reduction in M_3_R/*CHRM3* expression.

## 4. Muscarinic Receptor Agonism 

Many cellular functions are impacted by muscarinic receptor activation; however, the most impactful in CRC are likely to be those related to cell migration and invasion since the predominant cause of CRC morbidity and mortality is metastatic, stage IV disease. Hence, although M_3_R activation may stimulate CRC cell proliferation, the size of the primary tumor is only a concern as it may correlate with the likelihood of extraintestinal spread of disease. 

M_3_R overexpression per se does not account for its impact on CRC; the sources, availability, and concentrations of M_3_R agonists within the CRC microenvironment able to interact with M_3_R on neoplastic cells may play an equally important role. At present, only two *endogenous* ligands, acetylcholine (ACh) [25] and selected bile acids (BAs) [21,50], are known to activate muscarinic receptors. Regardless of whether M_3_R are activated by ACh or BAs, the propagation of downstream cell transduction stimulates CRC cell proliferation, resistance to apoptosis (survival), migration, and invasion [49]. Similar actions can be achieved by treating cells or mice with “designer” ACh mimetics, e.g., bethanechol, which are more resistant to hydrolysis by acetylcholinesterases.

Although ACh, a neurotransmitter, is typically produced by neurons [51], non-neuronal ACh can promote neoplasia [52,53,54,55,56] and for some cancers may even be the predominant source of ACh. In the tumor microenvironment, ACh may be produced by and released from enteric neurons, immunocytes, and CRC cells themselves [53,57]. Choline acetyltransferase (ChAT) plays an important catalytic role in the biosynthesis of both neuronal and non-neuronal ACh and its expression is reported in several organs and cancers, and is sometimes used as a surrogate marker of non-neuronal ACh production [25]. Using quantitative-PCR, Cheng et al. demonstrated ChAT expression and ACh production and release by H508, WiDr, and Caco-2 human colon cancer cells [56]. Notably, treating CRC cells with either selective or non-selective muscarinic receptor antagonists attenuated H508 colon cancer cell proliferation by 40% supporting the impact of endogenous production of ACh and autocrine effects. Inhibiting acetylcholinesterase activity increased H508 cell proliferation by as much as 2.5-fold, providing additional evidence that ACh can function as an autocrine growth factor for CRC [56].

These biological phenomena may have clinical consequences. Pheochromocytomas, uncommon neuroendocrine tumors that secrete excess catecholamines, may also produce excess ACh [58,59]. Despite previous endoscopic resection of a small focus of rectal cancer and vigilant surveillance, an elderly man with an unresectable pheochromocytoma experienced rapid recurrence of the rectal adenocarcinoma [60]. Analysis of tissue from the rectal carcinoma and pheochromocytoma revealed overexpression of M_3_R and ChAT, respectively [60]. For proof-of-principle, Rosenvinge et al. demonstrated that conditioned media from pheochromocytoma cells can stimulate the proliferation of H508 colon cancer cells, an action blocked by pretreating cells with the muscarinic receptor antagonist atropine [60]. These findings are consistent with the hypothesis that ACh released from the neuroendocrine tumor stimulated swift regrowth of remnant cells after endoscopic resection of the rectal cancer [59,60]. 

M_3_R can also be activated by non-traditional muscarinic ligands, such as selected BAs and their derivatives [61,62,63,64]. The potential for physiological BA signaling by this mechanism was discovered in 1998, when an interaction between BAs and M_3_R was observed in secretory gastric epithelial cells [50]. Structural similarities between BAs and cholesterol may explain the ability of the former to mimic the actions of the latter [61] and interact functionally with muscarinic receptor subtypes expressed selectively on Chinese hamster ovary (CHO) cells [61]; cholesterol is reported to act as an allosteric modulator of other GPCRs [65]. Further studies elucidated similarities between ACh and BA-induced post-muscarinic receptor signaling, primarily using H508 and HT-29 colon cancer cells. A prominent feature of these pathways is transactivation of epidermal growth factor receptors (EGFR) [63,66]; Cheng et al. showed that M_3_R and EGFR inhibitors and antibodies independently blocked the signaling and proliferative actions of BAs [64]. Thus, BA-induced colon cancer cell proliferation is M_3_R-dependent and mediated, in part, by transactivation of EGFR [64]. 

Post-mortem analysis of cecal contents from 19 persons without colorectal neoplasia revealed the presence of BA concentrations within the range capable of promoting colon cancer cell proliferation via M_3_R activation in vitro [67]; it is unknown whether fecal BA concentrations vary between persons with or without colonic neoplasia although, in rodents, increased concentration of fecal bile acids promote colon neoplasia. Mice fed a diet enriched in a secondary BA, deoxycholic acid, developed both precursor lesions of colon neoplasia and frank cancers [68,69]. Likewise, in mouse models of CRC, genetic ablation of a key intestinal BA transporter, ASBT, or of FGF15, a feedback inhibitor of hepatic BA synthesis, results in both increased fecal BA levels and promotion of colon neoplasia [68,69]. Notably, these findings may help to explain why consumption of a Western diet enriched in fats, beef, and processed meats that increase BA production may also increase the risk of CRC [70,71].

The effects of M_3_R agonism on the promotion of colon neoplasia are supported by in vivo evidence. In mice treated with azoxymethane (AOM), a procarcinogen that selectively induces colon neoplasia in rodents and mimics sporadic CRC in humans [72], M_3_R-deficiency dose-dependently reduced colon neoplasia [73]; similar effects were observed in *Apc^Min/+^* mice, a genetic model of colon neoplasia [74]. Conversely, Peng et al. showed that adding bethanechol, a non-selective muscarinic receptor agonist, to the drinking water of mice treated with azoxymethane significantly increased both the number and volume of colon tumors [75]. 

## 5. Targeting Muscarinic Receptors

As discussed above, M_1_ and M_3_ subtype muscarinic receptors are co-expressed in both normal and neoplastic intestinal epithelial cells [76]. Whereas, in azoxymethane-treated mice, ablating *CHRM3* expression attenuates colon neoplasia [72,73], ablating *CHRM1* did not significantly alter colon tumor number or size and, surprisingly, may have trended towards promoting colon neoplasia [76]. More importantly, concurrent ablation of both *CHRM3* and *CHRM1* negated the beneficial effects of *CHRM3* ablation [76]. These divergent effects of M_3_R and M_1_R on colon neoplasia in this animal model highlight the concern that to be effective, muscarinic receptor antagonists must be highly selective—off-target effects on other muscarinic receptor subtypes may either negate beneficial effects or, even more concerning, aggravate disease.

Amongst other factors, developing selective muscarinic receptor antagonists is challenging due to the highly similar orthosteric binding sites among the receptor subtypes [74]. Currently, no muscarinic receptor antagonists have been approved to treat cancer. However, in vivo studies using non-selective muscarinic antagonists such as scopolamine butylbromide demonstrated they can reduce intestinal tumor number and volume, albeit not as effectively as M_3_R gene (*CHRM3*) ablation [77]. Although scopolamine butylbromide does not cross the blood–brain barrier, besides being a non-selective muscarinic receptor antagonist, it is not specifically directed against intestinal muscarinic receptors and, therefore, its use may result in a wide variety of unwanted anti-cholinergic side effects [15]. As implied by the promising anti-neoplastic effects of genetic ablation of M_3_R/*CHRM3* [76,77], M_3_R targeting via highly selective antagonism is necessary to maximize efficacy and to prevent off-target effects on other muscarinic receptor subtypes, specifically M_1_R [76]. 

Given the widespread clinical use of M_3_R antagonists for other conditions, repurposing these medications for use as adjuvants to current CRC therapies should be considered and studied in clinical trials. In particular, the highly selective M_3_R antagonist, darifenacin, approved by the U.S. Food and Drug Administration (FDA) to treat genitourinary conditions, may be a viable option [78]. Notably, darifenacin, which is prescribed primarily for symptoms of an “overactive” bladder, arrested tumor progression in nude mice, further highlighting its potential for repurposed use [79]. Although muscarinic antagonists have been safely tolerated by most persons for many years, repurposing these medications warrants further investigation for potential dose-dependent toxicities at the levels and durations required to achieve anti-neoplastic effects. For example, case-control studies suggest long-term use of anti-cholinergic medications may be associated with an increased risk of dementia, particularly in those with pre-existing Parkinson Disease [80,81]. 

## 6. Targeting Matrix Metalloproteinases 

**Figure 1:**
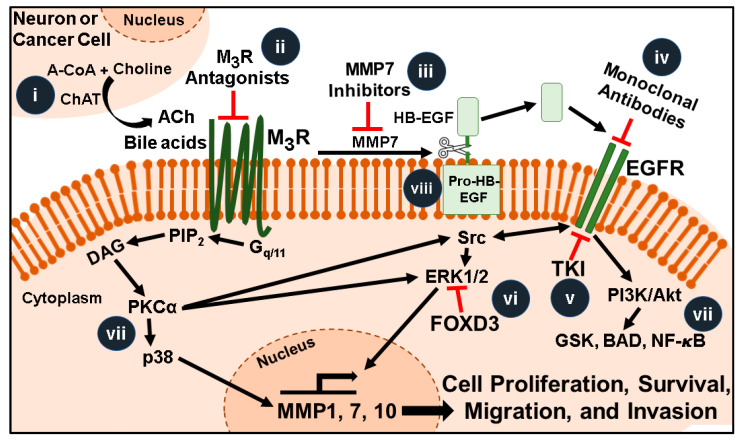
Muscarinic receptor signaling pathways and promising therapeutic targets for colorectal cancer. Acetylcholine (ACh, released from neurons, immunocytes, or adjacent cancer cells) and bile acids (BAs, in the fecal stream) activate M_3_ muscarinic receptors (M_3_R) overexpressed in colon cancer. Post M_3_R-signaling involves activation of protein kinase C-α (PKCα) and matrix metalloproteinase 7 (MMP7)-catalyzed release of heparin binding epidermal growth factor (EGF)-like growth factor (HB-EGF) which activates EGFR (EGFR-independent and -dependent signaling, respectively). Ultimately interactions between these complex signaling pathways induce transcription of genes that stimulate cell proliferation, survival, migration, and invasion. Amongst these genes, induction of matrix metalloproteinase-7 (*MMP7*) provides a “feed forward mechanism” to increase HB-EGF release. This illustration highlights features that are current or potentially future therapeutic targets: (**i**) Colon cancer cells can produce and release ACh, thus the molecules necessary for this process (e.g., choline acetyltransferase, ChAT) are potential therapeutic targets. (**ii**) Selective inhibitors of M_3_R can be repurposed to block the effects of ACh and bile acids. (**iii**) Inhibitors that selectively target MMP1 or MMP7 could block post-M_3_R signaling and degradation of the extracellular matrix. (**iv**) Monoclonal antibodies that block the EGFR binding site and (**v**) tyrosine kinase inhibitors that inhibit EGFR activation could be used in conjunction with selective M_3_R inhibitors to potentiate their inhibitory actions. (**vi**) FOXD3 overexpression or small molecular mimics block M_3_R- and EGFR-induced ERK activation. (**vii**) Highly selective inhibitors of the plethora of signaling molecules downstream of M_3_R and EGFR are in various stages of development. These include agents targeting Ras, ERK, PKC-α, and molecules comprising the apoptosis program (GSK, BAD, NF-ĸB). (**viii**) Pro-HB-EGF, a substrate for MMP7-activated release of HB-EGF and EGFR activation, is an unexploited therapeutic target.

In human CRC cell experimental models, muscarinic receptor agonists stimulate cancer progression via complex interacting signal transduction pathways involving both EGFR-dependent and -independent pathways (Figure 1). Downstream effectors of these pathways induce gene transcription programs resulting in cell proliferation, survival, migration, and invasion [49,66,82]. In particular, because of their critical role in degrading components of the extracellular matrix (ECM), M_3_R-induced matrix metalloproteinase (MMP) gene transcription is important for cell migration and invasion [83,84]; MMPs are key promoters of cancer progression [85]. MMPs are part of the metzincin family of metalloproteinases, comprised of 24 zinc-containing proteases that cleave components of the ECM in both health and disease [83,84]. Each class of MMPs, such as collagenases, gelatinases, stromelysins, and matrilysins, plays a different role [85,86]. Normally, MMP activity is closely regulated by tissue inhibitors, e.g., TIMPs [86]; in cancer, dysregulated MMP-TIMP expression may favor proteolysis [86], thereby contributing to cancer spread [87]. Giambernardi et al. reported abnormal expression of several MMPs in colon, breast, and prostate cancer cell lines [84]. MMP7 and MMP1 appear to play particularly important roles in CRC. 

In transgenic mouse models, MMP7 overexpression early in colon neoplasia promotes tumorigenesis; the converse is observed in MMP7-deficient mice [88,89]. Increased MMP7 expression in human CRC correlates with advanced disease and worse outcomes [90]. As a consequence of its ability to degrade ECM, MMP1, a collagenase, is a key player in colon cancer cell migration and metastasis [83,84], and its expression in human CRC is also associated with cancer progression, metastasis, and a poor prognosis [83,91]. Shiozawa et al. found absent MMP1 expression in colorectal adenomas, but its expression in 76% of CRC [73]. Enhanced MMP1 expression in invasive versus intramucosal CRC suggests increased expression of MMP1 in the earlier stages of tumor invasion, a similar pattern to that for increased M_3_R expression [73]. MMP1 expression correlates with infiltrative CRC, specifically with lymph node and liver metastasis [73]. MMP9 may also be associated with metastasis in CRC; high levels of both MMP1 and MMP9 expression in tumor-free mucosa correlated with TNM-stage and lymph node involvement [92]. 

Xie et al. showed that activating human colon cancer cell muscarinic receptors with ACh selectively induced expression of MMP 1, 7, and 10 [93], and Raufman et al. showed that treating CRC cells with atropine to block M_3_R activation or with a neutralizing anti-MMP1 antibody abolished ACh-induced cell invasion [83]. Nonetheless, despite their importance for CRC progression and promising in vitro and pre-clinical studies, two decades after initial clinical trials failed to show benefit for small molecule broad spectrum MMP inhibitors, targeting MMPs for cancer treatment remains challenging [94]. 

Initial therapeutic efforts were directed at developing inhibitors consisting primarily of a zinc-chelating moiety intended to target the active site of the MMP catalytic domain [95]. Approximately 50 such agents were tested and showed promising results in pre-clinical animal models—nevertheless, nearly all failed in clinical trials [95,96], largely due to off-target actions, metabolic instability, poor bioavailability, or dose-limiting side effects [97]. Disease stage may also have impacted discrepancies between effectiveness in pre-clinical studies and clinical trials; compared to murine models, subjects in clinical trials had more advanced disease [85,95,97]. Additionally, MMPs share structural similarity with the active site of other members of the metzincin family, resulting in broad and unexpected adverse effects [95,98]. Batimastat and marimastat, among the first MMP inhibitors tested in clinical trials [99,100], inhibit MMP 1, 2, 7 and 9, by binding to the active zinc site [101]. Although animal models demonstrated promising antitumor effects of batimastat [100,102], the clinical performance of batimastat, marimastat, and related compounds was disappointing and adverse events resulted in termination of clinical trials [99,103,104]; side-effects were largely attributed to off-target effects on molecules involved in vital cell functions such as cell–matrix interactions, cellular adhesion, and growth factor availability [105,106]. Another obstacle to developing MMP-targeted therapies is that expression of MMP substrates differs in mice and humans. For example, MMP7 activates intestinal α-defensin in murine but not human Paneth cells [85,107]. 

Advances in understanding structure-function relationships [95,108] led to renewed interest in selectively targeting the cancer-promoting actions of MMPs while retaining their important beneficial effects [109]. Notably, 10 of 24 MMPs have anti-tumorigenic and anti-inflammatory effects; inhibiting or downregulating their activity is likely to have adverse effects [109]. Using this information, investigators developed highly selective small-molecule inhibitors and antibodies against MMP 12 and 14 [110,111,112], monoclonal antibodies directed at the catalytic zinc-protein complex and enzyme surface conformational epitope of MMP 2 and 9 [15,113], and a highly selective compound, JNJ0966, that inhibits MMP9 activation [15,114]. JNJ0966, which interacts with a structural pocket distinct from the catalytic domain and proximate to the cleavage site of the MMP9 zymogen, attenuated disease severity in a mouse model of autoimmune encephalomyelitis, without altering the catalytic activity of MMP 1, 2, 3, or 14 [15,114]. These innovative approaches are likely to spur similar efforts to target other MMPs selectively. Nonetheless, considering the broad range of biochemical activities of MMPs, even selective inhibition may cause harm [109]. Alternative or adjunct strategies to avoid toxic systemic levels of MMP inhibitors may involve directed and targeted administration, likely as an adjunct to current chemotherapeutic regimens [15,85,94]. 

## 7. Epidermal Growth Factor Receptors 

As a consequence of MMP7-mediated release of an EGFR ligand HB-EGF, the epidermal growth factor receptor (EGFR), a receptor tyrosine kinase [66], may be transactivated after M_3_R activation (Figure 1) [16,115]. Consistent with these observations, inhibiting MMP activation with batimastat can block EGFR activation [116]. The prominent role of EGFR signaling in colon cancer cell [16] and its ability to modulate key hallmarks of cancer progression [117,118] has already stimulated the development of several anti-EGFR monoclonal antibodies and receptor tyrosine kinase inhibitors [118,119,120]. Nonetheless, M_3_R-stimulated EGFR transactivation may provide an additional therapeutic target [66]; it is likely that directly targeting MMP7, an enzyme that cleaves and releases HB-EGF from pro-HB-EGF, may augment the therapeutic benefits of other approaches (Figure 1). 

## 8. Additional Potential Therapeutic Targets Downstream of M_3_R 

Potential targets downstream of M_3_R and EGFR include RAS, BRAF, and components of mitogen activated protein kinase (MAPK) signaling. Recently, Liu et al. studied a well-known transcriptional repressor associated with several cancers, forkhead box D3 (*FOXD3*). Their findings revealed *FOXD3* knockdown considerably enhanced the proliferation and invasiveness of human colon cancer cells [121]. *FOXD3* knockdown activated a key signaling pathway in human colon cancer cells, EGFR-Ras-Raf-MEK-ERK [121]. While the exact mechanism of *FOXD3* association with the EGFR-Ras-Raf-MEK-ERK signaling pathway is unclear, enhancing the expression of *FOXD3* or promoting its activation may have potential as a therapeutic strategy [15,121]. Investigators have also attempted direct BRAF inhibition, especially in BRAF mutant mCRC [122]. Unfortunately, drugs such as vemurafenib, an oral single-agent *BRAF*^V600E^ inhibitor, have not shown meaningful in vivo activity. The limited activity of single-agent BRAF inhibitors appears due primarily to mechanisms of resistance and feedback regulation intrinsic to the RAS/RAF/MAPK signaling pathway [122]. It is noteworthy that over 30 years following the discovery of the role of *KRAS* in cancer growth and development, no drugs targeting *KRAS* are currently in clinical trials [123,124]. These challenges are due in large part to RAS being the most frequently mutated oncogene across all malignancies, the pervasiveness of compensatory *RAS*-mediated signal transduction feedback loops, and signaling elicited by oncogenic gain-of-function mutations [123].

As demonstrated in human embryonic kidney cells, MAPK activation was attenuated after inhibiting Src, a non-receptor protein tyrosine kinase protooncogene whose activation supports cell survival [125]. Src regulates multiple pathways and is overexpressed in CRC where its activity promotes metastasis and may contribute to chemotherapy resistance [126]. Through its kinase activity, Src potentiates the effects of EGFR activation [127,128,129]. In H508 colon cancer cells, inhibiting Src attenuated ACh- and EGF-induced ERK1/2 phosphorylation (activation), identifying Src as a key link between M_3_R-induced transactivation of EGFR and the subsequent downstream activation of MAPK (ERK1/2) in CRC [66]. Moreover, warranting consideration is a related pathway involving receptors for corticotrophin-releasing factor-2 (CRF2), an important neuromodulator of stress. Crosstalk between CRF2 and M_3_R signaling augments colon cancer cell migration, invasion, and other attributes promoting cancer progression [130]. Pelissier-Rota et al. revealed a novel intercellular circuit whereby CRF2 agonists in conjunction with ACh-induced activation of M_3_R and a feedback loop resulting in additional release of a CRF2 agonist, urocortin-3, modulates activation of Src/Erk and focal adhesion kinase (FAK). Besides unveiling unique crosstalk between muscarinic receptors and CRF2, interactions between these signal transduction pathways may alter colonic mucosal barrier function, inflammation, and the risk of developing colitis-associated cancer, particularly in those with inflammatory bowel diseases [130].

Muscarinic receptor activation promotes protein biosynthesis, thereby enhancing colon cancer cell proliferation by ERK1/2-mediated pathways. In SNU-407 colon cancer cells, Park et al. explored how modulating muscarinic receptor activity affected eukaryotic translation elongation factor 2 (eEF2), the protein responsible for ribosomal translocation [131,132,133]. Treatment with muscarinic receptor agonists reduced eEF2 phosphorylation, thereby inhibiting its activity and the rate of translation, effects blocked by pre-treatment with atropine [134,135,136]. Treating cells with a potent MEK1/2 inhibitor (U0126) or protein kinase C inhibitor, GF109203X, decreased carbamylcholine-induced eEF2 dephosphorylation. These findings provide further evidence of the importance of MEK1/2-ERK1/2 and PKC signaling downstream of muscarinic receptor activation and implicate a novel role for eEF2 dephosphorylation, another potential therapeutic target.

Notably, chlorpyrifos (CPF) and other widely used organophosphate insecticides associated with exposure-dependent cancer risk activate signaling pathways similar to those following muscarinic receptor activation [137]; in H508 colon cancer cells, CPF increased EGFR phosphorylation and downstream activation of ERK1/2 [138]. These effects were attenuated by treatment with U0126, a MEK1/2 inhibitor, and AG-1478, an EGFR tyrosine kinase inhibitor [138]. As organophosphates act by inhibiting acetylcholinesterase activity, thereby increasing ACh levels, these findings suggest that interventions that block the pro-neoplastic effects of muscarinic receptor activation can also be leveraged to block the actions of this family of carcinogens that hijack components of the same signaling mechanisms.

## 9. Conclusions and Future Directions

The illustration depicted in Figure 1 summarizes the current state of knowledge regarding key elements of muscarinic receptor signaling and therapeutically targetable nodes in CRC. These nodes include the machinery for ACh production in the tumor microenvironment, selective inhibitors of M_3_R activation and MMPs, like MMP7 which mediates release of HB-EGF and activation of EGFR, and a host of key downstream signaling molecules. In addition to advances in selective drug design and development and preclinical and clinical trials of new and repurposed pharmaceuticals the conundrum underlying the apparent opposing actions of M_3_R and M_1_R in colon cancer progression must be solved as this may reveal an entirely novel, potentially exciting therapeutic strategy [76]. Given the tumor cell heterogeneity within colon cancers and redundant signaling pathways, it is likely that combinatorial therapy will be required to impede the complex interacting signal transduction pathways shown in Figure 1 and provide meaningful therapeutic gains for patients with advanced CRC.
